# Wearing a KN95/FFP2 facemask induces subtle yet significant brain functional connectivity modifications restricted to the salience network

**DOI:** 10.1186/s41747-022-00301-0

**Published:** 2022-10-10

**Authors:** Sven Haller, Marie-Louise Montandon, Cristelle Rodriguez, Panteleimon Giannakopoulos

**Affiliations:** 1 CIMC - Centre d’Imagerie Médicale de Cornavin, Geneva, Switzerland; 2 Department of Rehabilitation and Geriatrics, Geneva University Hospitals and University of Geneva, Geneva, Switzerland; 3 Department of Psychiatry, Faculty of Medicine, University of Geneva, Geneva, Switzerland; 4 Division of Institutional Measures, Medical Direction, University Hospitals of Geneva, Geneva, Switzerland

**Keywords:** COVID-19, Default mode network, Magnetic resonance imaging, N95 respirators, Masks

## Abstract

**Background:**

The use of facemasks is one of the consequences of the coronavirus disease 2019 (COVID-19) pandemic. We used resting-state functional magnetic resonance imaging (fMRI) to search for subtle changes in brain functional connectivity, expected notably related to the high-level salience network (SN) and default mode network (DMN).

**Methods:**

Prospective crossover design resting 3-T fMRI study with/without wearing a tight FFP2/KN95 facemask, including 23 community-dwelling male healthy controls aged 29.9 ± 6.9 years (mean ± standard deviation). Physiological parameters, respiration frequency, and heart rate were monitored. The data analysis was performed using the CONN toolbox.

**Results:**

Wearing an FFP2/KN95 facemask did not impact respiration or heart rate but resulted in a significant reduction in functional connectivity between the SN as the seed region and the left middle frontal and precentral gyrus. No difference was found when the DMN, sensorimotor, visual, dorsal attention, or language networks were used as seed regions. In the absence of significant changes of physiological parameter respiration and heart rate, and in the absence of changes in lower-level functional networks, we assume that those subtle modifications are cognitive consequence of wearing facemasks.

**Conclusions:**

The effect of wearing a tight FFP2/KN95 facemask in men is limited to high-level functional networks. Using the SN as seed network, we observed subtle yet significant decreases between the SN and the left middle frontal and precentral gyrus. Our observations suggest that wearing a facemask may change the patterns of functional connectivity with the SN known to be involved in communication, social behavior, and self-awareness.

## Key points

- Facemasks have no significant effect on low-level cognitive networks.
- Facemasks have subtle yet significant effect on high-level salience network interaction.
- Facemask-related fMRI changes are not the consequence of respiration/heart rate modifications.

## Background

Wearing a facemask in professional settings was usually restricted to medical personnel, and surgical facemasks were typically used. The COVID-19 pandemic changed this reality, including the wearing of facemasks in daily life interactions and in magnetic resonance imaging (MRI) scanning facilities. This practice is widely accepted and well tolerated, thought to be effective for infection prevention [1].

In principle, wearing a facemask should not limit sensorial processing or high-level abilities such as thinking, reading, and writing. However, by impacting social interaction, facemasks modify the neural responses to recognition of facial cues but also pivotal human abilities serving our daily behavior such as emotion recognition, perceived closeness, trust attribution, and even re-identification of unmasked faces [2–6]. Unlike behavioral paradigms, little is known about the effect of wearing a facemask on brain activation. The two available studies indicated that this practice has a subtle but still significant effect on cerebral blood flow and oxygen saturation but also blood oxygenation level dependent (BOLD) baseline signals [7, 8]. In a series of 13 young individuals wearing a FFP2 facemask, Fischer et al. [7] reported a 6.5% increase of cerebral blood flow and a 0.9% increase of oxygen saturation measured by transcranial hybrid near-infrared spectroscopy. In eight middle-aged subjects with a classical surgical facemask, Law et al. [8] indicated an altered BOLD baseline signals with a minimal impact on task-related activation.

To our knowledge, no previous study explored the effect of wearing facemask in at rest functional connectivity. We opted for focusing on two main networks involved in complex prosocial behaviors, the salience network (SN) and default mode network (DMN) [9]. Early studies showed that the patterns of functional connectivity of these networks are critically involved in various psychiatric disorders, including anxiety disorders, autism, schizophrenia, bipolar disorders, and dementias [10–15]. More recently, the interplay between these networks has been involved in the successful performance of social-cognitive tasks toward a close other [16] as well as mentalizing performances [17].

In this study, we tested the hypothesis that wearing a facemask could induce changes in the at rest functional connectivity of these networks that may ultimately impact on our social interaction abilities.

## Methods

### Participants

This prospective study was approved by the Ethics Committee of the University.

Hospitals and University of Geneva, Switzerland. The study was in accordance with the Declaration of Helsinki, and all participants gave written informed consent. We included 24 community-dwelling cases recruited via advertisements in local media. One case was excluded from further analysis due to the incidental finding of an enlarged ventricular system. The final sample included 23 community-dwelling young men aged 29.9 ± 6.9 years (mean ± standard deviation) recruited via advertisements in local newspapers and social media. The following exclusion criteria were applied: (i) presence or history of a chronic psychiatric disorder (psychosis, bipolar disorder); (ii) history of loss of consciousness lasting longer than 30 min; (iii) history of head injury or post-concussion symptoms; (iv) history of auditory or visual deficits, seizure, and neurological disorders; and (v) regular use of psychotropic medications or alcohol. The exclusion of acute psychiatric disorders was confirmed by the Mini Neuropsychiatric Interview [18].

### fMRI acquisition protocol

The study was performed during the COVID-19 pandemic (from 2021/01 to 2021/05). All participants wear facemasks before coming to the MRI facility. For the fMRI experiment, all participants had the same KN95/FFP2 (KN95, China GB2626-2006; FFP2, Europe EN 149–2001) facemask (a commercial model without metal to be safe in the 3-T MRI environment). We used a crossover design: half of the participants had first the mask (MASK) and then no mask (NOMASK), the other half had the inverse order. The entire MRI scanning lasted approximatively 1 h. We made sure that participants had the facemask on for 10 min before the start of MASK, and no facemask for 10 min before NOMASK. To avoid potential bias of the resting fMRI results due to basic physiologic parameters, we monitored breathing and heart rate during the fMRI runs.

Images were acquired using a 3-T MRI scanner (Magnetom Prisma, Siemens Healthineers, Erlangen, Germany) at Campus Biotech Geneva (https://www.campusbiotech.ch/). An echo-planar resting-state fMRI echo-planar sequence and a three-dimensional T1-weighted sequence were acquired, the latter used for spatial normalization and registration. Technical parameters of both sequences are listed in Table 1. Each participant performed two runs in a crossover design, once with and once without a KN95/FFP2 facemask. Heart rate and respiration data were collected continuously and simultaneously for resting-state functional imaging using *BIOPAC systems (*https://www.biopac.com/research/*).*

**Table 1:** Magnetic resonance imaging (MRI) sequence parameters

Sequence parameters of the resting-state fMRI protocol	
Nr. of slices	66
Slice thickness (mm)	2.0
Voxel size (mm^3^)	2.0 × 2.0 × 2.0
Repetition time (ms)	1,000
Echo time (ms)	32
Flip angle (°)	50
Field of view (mm)	224
Acquisition time (min)	7:11
3DT1 sequence parameters	
Nr. of slices	208
Slice thickness (mm)	1.0
Voxel size (mm^3^)	1.0 × 1.0 × 1.0
Repetition time (ms)	2,300
Echo time (ms)	2.26
Flip angle (°)	8
Field of view (mm)	256
Acquisition time (min)	4:44

### Image analysis

Image analysis was performed using the CONN toolbox version 20b (www.nitrc.org/projects/conn, RRID:SCR_009550).

#### fMRI data preprocessing

fMRI data analysis was performed using the standard processing steps in the CONN toolbox as described in detail previously [19]. Briefly, the data processing included motion correction, spatial filtering, denoising, and transformation into NMI standard space using the individual 3DT1 brain sequence for anatomic spatial registration. The fMRI data were then parcellated using the functional atlas included in the CONN toolbox. The functional regions of interest (ROIs) considered here included default mode network (DMN), sensorimotor network, visual network, SN, dorsal attention, frontoparietal, and language networks as well as cerebellum (control area). Each ROI was divided into subregions of interest. As an example, the relevant subregions for the SN were Salience ACC (anterior cingulate gyrus), Salience Alnsulal (left anterior insula), Salience Alnsular (right anterior insula), Salience RPFCl (left rostral prefrontal cortex), Salience RPFCr (right rostral prefrontal cortex, Salience SMGI (left supramarginal gyrus), and Salience SMGr (right supramarginal gyrus). Then, the functional time series were analyzed using the following two steps.

#### ROI-to-ROI analysis

The ROI-to-ROI connectivity metrics characterize the connectivity between all pairs of ROIs among a predefined set of regions. This atlas notably includes predefined set of regions for all of the networks considered. This ROI-to-ROI analysis examines the functional connectivity between each pair of ROIs, for example, that between DMN and SN. This pairwise ROI-to-ROI connectivity analysis was compared between MASK *versus* NOMASK conditions.

#### Seed-to-voxel analysis

The seed-based connectivity analysis aims to investigate functional properties from preselected seed regions. As seed regions, the same functional atlas-based regions were used as described above focusing on SN, DMN, visual, dorsal attention, frontoparietal, and language networks. Similar to the ROI-to-ROI analysis described above, a pairwise comparison between MASK *versus* NOMASK conditions was performed.

Both analyses used the same predefined atlas regions as ROI/Seed. The ROI-to-ROI only investigates functional connectivity between predefined ROIs but cannot detect functional connectivity changes in other (not predefined) regions. In contrast, seed-to-voxel analysis is able to detect functional connectivity changes of the preselected seed ROI to any unselected voxel of the brain without reference to predefined functional anatomic regions.

### Statistical analysis

Data are presented as mean ± standard deviation. Multiple-comparison correction was applied using the false discovery rate (FDR) [20]**.** Concerning the seed-to-voxel analyses, parametric statistics (Gaussian Random Field theory) cluster threshold: *p* < 0.05 cluster-size p-FDR [20] corrected; voxel threshold: *p* < 0.001 p-uncorrected.

### Declarations

A first preprint non-peer-reviewed version of this paper has been published in ResearchSquare https://www.researchsquare.com/article/rs-1171603/v1.

## Results

There were no differences in heart rate or respiration rate between MASK and NOMASK conditions. The values provided by the MRI system *BIOPAC* are unitless values (for respiration frequency and pulse rate) and were for respiration 4.54 ± 1.55 (MASK) *versus* 4.88 ± 1.54 (NOMASK), *p* = 0.460, and for pulse 0.12 ± 0.01 (MASK) *versus* 0.12 ± 0.01 (NOMASK), *p* = 0.225.

**Fig. 1:**
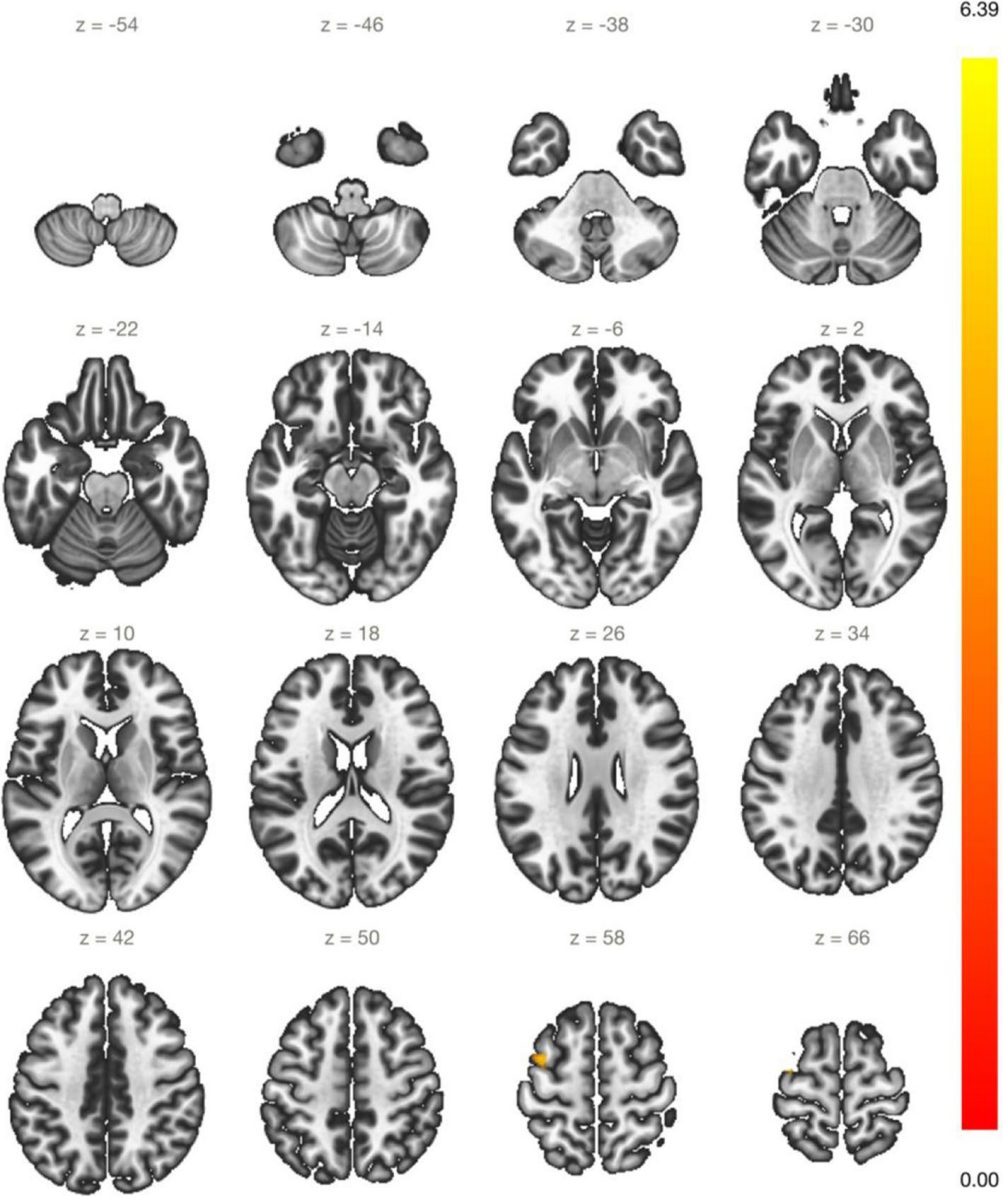
The functional connectivity seed-to-voxel (region of interest) analysis using the salience network as seed region resulted in one significant cluster in left middle frontal and precentral gyrus (threshold: *p* < 0.05 cluster-size; *p*-false discovery rate corrected). See Table 2

We found no significant direct effect of wearing a facemask within the established resting state networks, including the SN, DMN, sensorimotor, visual, dorsal attention, or language networks. The resting fMRI seed-to-voxel analysis using the SN as the seed region revealed a significant reduction in a cluster in the left middle frontal and precentral gyrus for MASK *versus* NOMASK conditions (Fig. 1, Table 2).

**Table 2:** Seed-to-voxel functional connectivity for the salience network (SN) as seed region

Cluster (x,y,z)	Size	Size *p*-FWE	Size *p*-FDR	Size *p*-unc	Peak *p*-FWE	Peak p-unc
− 30 + 02 + 64	151	0.017484	0.012910	0.000679	0.101036	0.00001

The functional connectivity SEED-to-VOXEL (region of interest) analysis using the SN (salience network) as seed region resulted in one significant cluster of 151 voxels, as illustrated in Fig. 1. The details of this cluster are listed in this table. *P-FWE*, *p* value familywise error corrected; *P-FDR*, *p* value false discovery rate corrected; *P-unc*, *p* value uncorrected

This cluster of 151 voxel includes 102 voxels (68%) covering 3% of atlas.MidFG I (middle frontal gyrus left), 21 voxels (14%) covering 0% of atlas.PreCG I (precentral gyrus left), and 28 voxels (19%) covering 0% of atlas.not-labeled

**Fig. 2:**
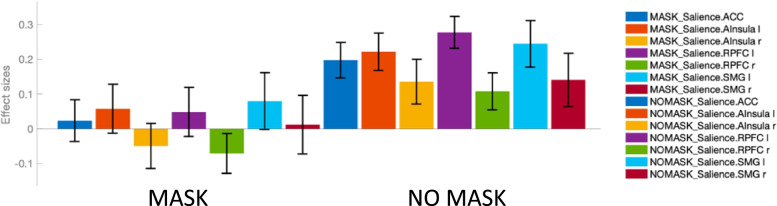
Detailed analysis of the significant cluster identified of the seed-to-voxel analysis using the salience network (SN) as seed region (see Fig. 1). The functional connectivity between the SN and this cluster was significantly lower (*F*([7 16]) 8.60; *p*-false discovery rate < 0.001) for MASK *versus* NOMASK conditions. Of note, this effect was present for all seven subregions of the SN

The details of this significant cluster are illustrated in Fig. 2. The functional connectivity between the SN as seed region and this cluster was significantly (*p*-FDR < 0.001) lower for MASK *versus* NOMASK conditions. This effect was present for all seven subregions of the SN.

There were no significant group differences using the DMN or sensorimotor, visual, dorsal attention, or language networks as seed regions.

## Discussion

The present findings indicate that the effect of wearing a tight FFP2/KN95 facemask on brain functional connectivity was significant using the SN but not the DMN, sensorimotor, visual, dorsal attention, or language networks as seed regions. Basic physiological effect of altered breathing cannot be at the origin of the observed differences. This assumption is supported by two observations. First, we did not observe significant differences in the measured heart rate and respiration rate. Second, if the effect was a basic effect due to respiration, this effect should be present in all networks, including basic-level and high-level networks, but we observed a change in fMRI connectivity only in high-level SN.

The only previous study available in this field [8] assessed the effect of wearing a facemask on functional MRI but with different approach and objectives. This study assessed task-related fMRI during a basic sensory-motor task designed to activate visual, auditory, and sensorimotor cortices in eight middle-aged participants. The authors reported an altered BOLD baseline signals with no impact on task-related activation of sensorimotor areas. In our study, resting-state fMRI analysis in 23 cases focused on functional connectivity of higher-level resting networks. We also report no significant effect of wearing facemask on the functional connectivity of lower-level sensorimotor or visual networks. In agreement with our a priori hypothesis, the effect of this practice was confined to an interaction between the SN as the seed region and the left middle frontal and precentral gyrus. Although subtle, this effect concerned all of the SN subregions and was statistically significant.

The clinical significance of this finding remains unclear. Altered functional connectivity of the SN with frontal and precentral areas was reported as part of a more global cortical disconnection in a variety of neuropsychiatric disorders, including psychosis, poststroke depression, and attention deficit syndrome [21–23]. In young controls, the connectivity between the SN and left frontal as well as precentral areas is thought to be crucial not only for episodic memory skills [24] but also for working memory activation [25]. Our observations should be interpreted in conjunction with the recent report by Fischer et al. [7] who reported a subtle but still significant increase of cerebral blood flow and oxygen saturation when using the same type of facemask. Taken together, these observations suggest that wearing a FFP2/KN95 facemask may lead to compensatory increase of brain metabolism and deficits of functional connectivity in the SN. This latter is involved in the detection of environmental stimuli and mediates the transition from the DMN to the central executive network [9, 26]. Whether these changes are clinically silent or may predispose individuals to fail in highly demanding situations merits further consideration. Our observations should be viewed as a first step towards a more detailed analysis of facemask wearing repercussions in complex environments. In particular, the study of facemask wearing effect on brain activation in social interaction and mentalization paradigms, two main functions subserved by the SN, is a critical issue to address in order to get insight into the clinical significance of our work.

Some limitations should be considered when interpreting the present findings. To avoid the well-documented gender-related differences in functional connectivity [27], we included only male participants in this study. Although this procedure makes sense given the small series of cases that accepted this constraining experimental design, it naturally limits the generalizability of the present findings. Moreover, we deliberately used a tight FFP2/KN95 facemask, which many people prefer in the context of the COVID-19 pandemic and are requested by most airline companies. We would expect smaller effects on brain functional connectivity when using less tight surgical facemasks, which was the standard facemask in the field of medicine before the COVID-19 pandemic. Third, the presence of physiological noise could impact the quality of fMRI data. Last but not least, the changes in functional connectivity reported may vary as a function of the length of the period that subjects wear the masks. Since no changes in physiological parameters related to wearing a facemask were observed in this series, it is highly likely that the reported result is probably stable over time. However, the present experimental design does not allow for addressing this hypothesis.

Future studies with various types of masks in mixed samples including several time points of wearing facemask and using ad hoc activation paradigms that involves the SN will make it possible to complete these at rest observations, gaining better insight into the impact of facemask wearing on brain function.

## Data Availability

Data availability is limited by local data sharing regulations.

## Abbreviations

BOLD: Blood oxygenation level-dependent
COVID-19: Coronavirus disease 2019
DMN: Default mode network
FDR: False discovery rate
fMRI: Functional magnetic resonance imaging
ROI: Region of interest
SN: Salience network
